# Recent Advances in the Pathogenesis of Syndromic Autisms

**DOI:** 10.1155/2009/198736

**Published:** 2009-06-21

**Authors:** A. Benvenuto, B. Manzi, R. Alessandrelli, C. Galasso, P. Curatolo

**Affiliations:** Pediatric Neurology Unit, Department of Neuroscience, “Tor Vergata” University of Rome, 00133 Rome, Italy

## Abstract

*Background*. Current advances in genetic technology continue to expand the list of medical conditions associated with autism. Clinicians have to identify specific autistic-related syndromes, and to provide tailored counseling. The aim of this study is to elucidate recent advances in autism research that offer important clues into pathogenetic mechanisms of syndromic autism and relevant implications for clinical practice. *Data Sources*. The PubMed database was searched with the keywords “autism” and “chromosomal abnormalities,” “metabolic diseases,” “susceptibility loci.” *Results*. Defined mutations, genetic syndromes, and metabolic diseases account for up to 20% of autistic patients. Metabolic and mitochondrial defects may have toxic effects on the brain cells, causing neuronal loss and altered modulation of neurotransmission systems. Alterations of the neocortical excitatory/inhibitory balance and perturbations of interneurons' development represent the most probable pathogenetic mechanisms underlying the autistic phenotype in Fragile X-Syndrome and Tuberous Sclerosis Complex. Chromosomal abnormalities and potential candidate genes are strongly implicated in the disruption of neural connections, brain growth, and synaptic/dendritic morphology. *Conclusion*. Metabolic testing may be appropriate if specific symptoms are present. High-resolution chromosome analysis may be recommended if a specific diagnosis is suspected because of obvious dysmorphisms. Identifying cryptic chromosomal abnormalities by whole genome microarray analysis can increase the understanding of the neurobiological pathways to autism.

## 1. Introduction

Autism and related autism spectrum disorders (ASDs) are heterogeneous neurodevelopmental disorders behaviorally defined by significant deficits in social interaction and communication and by the presence of restricted interests and repetitive behaviors [1]. Autism Disorder (AD) comorbidy with seizures and mental retardation occurs in up to 30% and in 80% of autistic patients, respectively [2]. Its pathogenetic substrates are still largely unknown. Despite strong familial components, clinical complexity has posed a major challenge to our understanding of autism pathogenesis. Genetically, the picture is complicated by significant interindividual heterogeneity, numerous contributing loci, and multiple genes and gene-environment interactions [3]. Several lines of evidences strongly support a prenatal onset for developmental abnormalities later leading to autism [4]. Autism in its very broad spectrum of severity is known to have many different etiologies. In the last few years, significant progresses have been made in comprehending the causes of autism, and their multiple impacts on the developing brain [5]. The primary goals of treatment are optimizing the quality of life and minimizing the impairment due to the core symptoms of autism [6].

In this article we discuss current understanding of the pathogenesis of syndromic autism and the multiple pathways responsible for the ASD phenotype.

## 2. Metabolic Diseases

Several inborn errors of metabolism, including phenylketonuria, biotinidase deficiency, disorders of cerebrospinal fluid (CSF) neurotransmitters such as deficiencies of folic acid, Smith-Lemli-Opitz syndrome (SLOS), creatine deficiency syndromes, metabolic purine disorders, have an autistic phenotype [7]. A better understanding of some of them has implications both for discovery of the pathophysiologic underpinnings of the disorder and for the development of effective interventions.

In untreated children affected by phenylketonuria, the high levels of phenylalanine may have toxic effects on the brain cells, causing reduction of myelin, neuronal loss, and decreased levels of interneuronal connections [8]. Hyperphenylalaninemia also competes with the absorbition of other amino acids and consequently lower tyrosine and tryptophan concentrations can result in a low production of dopamine and serotonin in the prefrontal cortex [9].

In some of the most severe metabolic diseases, like adenylosuccinase deficiency or creatine deficiency syndromes, neurological and behavioral symptoms are probably not caused by deficiency of metabolites, but are more likely due to the toxic effects of the accumulating substances on the brain [8]. A direct role in modulation of dopaminergic and serotoninergic neurotransmission systems and axonal guidance has been hypothesized for the adenosine deaminase deficiency as pathologic mechanisms for the development of altered pathways involved in autistic symptoms [10]. The role of mitochondrial disorders has been revitalized by the association between autism and variants of the SLC25A12 gene, which encodes the predominant isoform of the mitochondrial aspartate (asp)/glutamate (glu) carrier (AGC) in brain [11]. Altered Ca2+ homeostasis is responsible for boosting AGC activity, mitochondrial metabolism, and, to a more variable degree, oxidative stress in autistic brains [12]. Based on our clinical experience, routine metabolic screening studies should be used on a case by-case basis, in the presence of the autistic regression, or suggestive clinical findings, such as lethargy, cyclic vomiting, early onset seizures, dysmorphic features, mental retardation with neurologic deficits, unexplained immune deficiency or unexplained hemolytic anemia, hyper- or hypotonia, self-mutilation, and muscle weakness [13]. Table 1 summarizes the main clinical features, diagnostic test and therapeutic options of the metabolic diseases most frequently associated with ASD.

## 3. Epilepsy and Regressive Autism

The relationship among epilepsy, autism, and regression is a poorly understood and controversial subject. There are several epilepsy syndromes in which regression of language, cognition, and behavior may lead to clinical manifestations that overlap with the behavioral syndrome of autism, such as infantile spasms, slow spike-wave discharges during sleep, and focal centrotemporal spikes. An epileptic disorder must be considered in all children with a low functioning ASD, especially when a history of regression and electroencephalogram (EEG) epileptic abnormalities is present [14]. Severe epileptiform abnormalities may permanently alter the critical synaptogenesis by strengthening synaptic contacts that should have been naturally pruned [15]. Cognitive functions decline in those patients who have early-onset EEG abnormalities and a prolonged active phase of continuous spike-and-wave discharges during sleep [16]. In children with autistic regression, there is no evidence that treatment of the seizures or of the interictal epileptiform activity makes a difference in regard to the outcome of the language and social deficits. Because the relationship between autism, epilepsy, and regression is complex, the clinician's index of suspicion for epilepsy should be high, and treatment of the epilepsy should be pursued when necessary.

Although the pathogenetic link between autism and epilepsy is poorly understood, the existence of altered Ca2+ signaling in ASD and the bioelectrical instability resulting from mutations of the L-type voltage-gated Ca2+ channels associated to autism may account for the high prevalence of seizures and/or EEG abnormalities present among autistic individuals [17].

## 4. Genetic Diseases Associated with Autism

Single gene defects and chromosomal abnormalities may account for approximately up to 10% [18] of individuals with autism, and the fraction is likely to be higher when microarray comparative genome hybridization is used [19]. Table 2 summarizes the most frequent genetic syndromes and cytogenetic abnormalities associated with autism.

### 4.1. Fragile X Syndrome

Abnormalities in long-term synaptic plasticity of excitatory synapses and in baseline synaptic connectivity may be the underlying neurological substrate of autism associated with FXS [20, 21]. Alterations in the neocortical excitatory/inhibitory balance as well as abnormal neural synchronization have been also reported in mouse model of FXS [22], resulting in hyperexcitability of neocortical circuits. An immature dendritic morphology may also increase susceptibility to epilepsy and anxiety in FXS patients [23].

### 4.2. Tuberous Sclerosis Complex

Tuberous sclerosis complex (TSC) is an inherited disorder resulting from mutations in one of two genes, TSC1 (Hamartin) and TSC2 (Tuberin), characterized by benign hamartomatous tumors that involve multiple organ systems. It is commonly associated with neuropsychiatric complications like epilepsy, mental retardation, autism, and other behavioral problems. Seizures can be present in the first year of life and up to one third of children develop infantile spasms. Neurobehavioral phenotypes in TSC may arise from perturbations of interneurons development, which can selectively impact frontal and parietal areas [24]. TSC2 gene localized on 16p13.3 locus encodes for tuberin, a protein highly expressed in frontal regions [25]. Furthermore, several studies have described the TSC 1 locus 9q34 as an important region of vulnerability for the developmental of autism. A loss of a single TSC1 gene copy in mice is sufficient to perturb cytoskeletal dynamics and dendritic spine structure, highlighting generalized neurotrophic roles for these genes, in addition to cell growth regulation. Circuitry alterations are the possible biological substrate of autism associated with TSC.

## 5. Chromosomal Abnormalities

A wide number of cytogenetic abnormalities have been described [26], particularly in the low functioning autistic population with dysmorphic features [27].

### 5.1. Chromosome 15

Chromosomal rearrangements in 15q11-15q13 region might be the most frequent cytogenetic abnormality in ASD [34], accounting for 1–2% of patients. A chromosome 15 phenotype II, characterized by ataxia, language delay, epilepsy, mental retardation, repetitive movement disorders, and facial dysmorphic features, has been described in individuals with chromosome 15 duplications [35]. Within the 15q11–15q13 locus, gamma-aminobutyric acid A receptor beta 3 (*GABRB3)*, an inhibitory neurotransmitter receptor, are currently thought to be central likely to play a significant role in the development of ASD, due to its role in the neuronal inhibition and its expression in early development [36]. This finding is particularly interesting in light of the high incidence of seizures and EEG abnormalities in autistic patients.

### 5.2. Chromosome 7

Two of the loci most commonly associated with ASD by genetic linkage studies [37, 38] (7q22 and 7q31 regions) contain several genes implicated in the pathogenesis of autism. The RELN gene, found within the 7q22 region, has a pivotal role in neuronal migration and prenatal development of neural connections, [39, 40] and is potently inhibited by toxic substances, such as organophosphates [41].

Increased risk for autism can be also linked to a functional polymorphism in the MET gene, found within the 7q31 locus [42], which plays a role into development of the cerebral cortex and cerebellum. The Williams-Beuren syndrome (WBS) region (7q11.23) also contains several genes associated with impairment in language and social interaction [43–45], suggesting the existence of a specific subgroup of autistic patients, characterized by dysmorphic features, mental retardation, language delay, congenital heart disease, and hypersensitivity to sound.

### 5.3. Chromosome 16

An association between a larger microdeletion on 16p11.2 and a syndrome that included developmental delay and distinct facial appearance (hypertelorism, a broad nasal bridge and a broad nasal tip with a prominent columella, a short philtrum, long ears, a large mouth) has been described [46–48]. The chromosomal region 16p11.2 also encompasses the PRKCB1 locus, an interesting gene previously found associated with autism [49], and expressed in the CNS, the immune system, the digestive tract, and the kidney. A recent study has described an association between PRKCB1 and an enhanced urinary peptide excretion rate [50].

### 5.4. Chromosome 2

Deletions involving 2q37 have been observed in more than 70 individuals with autism, mental retardation, and dysmorphic features (prominent forehead, depressed nasal bridge, dysmorphic ears and nose, short stature, and short hands and feet) [51, 52]. Three different breakpoints of 2q37 (2q37.1, 2q37.2, 2q37.3) have been analyzed to clarify the genotype-phenotype relationships associated with different terminal deletions [53], and several candidate genes for autism have been identified in 2q37.3 band [54]. Furthermore, a correlation between autism and a de novo cryptic deletion of chromosome 2p25.2 has been described [55]. The interaction between potential candidate genes that are expressed on these loci may explain the phenotypical heterogeneity and the spectrum of neuropsychological deficits associated with 2q37 and 2p25.2 deletion syndromes.

Other regions implicated in the ASDs with possible candidate genes are summarizes in Table 3.

## 6. Pathogenetic Pathways

**Figure 1:**
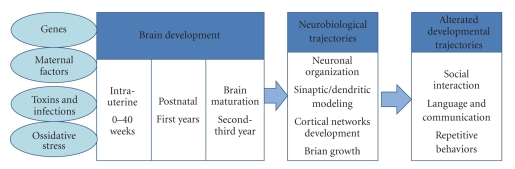
*Genetic and epigenetic factors involved in the pathogenesis of autism*. Interactions between multiple genes and environmental factors, such as intrauterine infections, alcohol/toxins exposure, and obstetrical suboptimality, can influence intrauterine and early postnatal brain development and disrupt crucial neurobiological pathways, from neuronal migration and cortical organization to synaptic and dendritic conformation, resulting in alterations of neurobehavioral trajectories that are involved in the pathogenesis of ASD.

**Figure 2:**
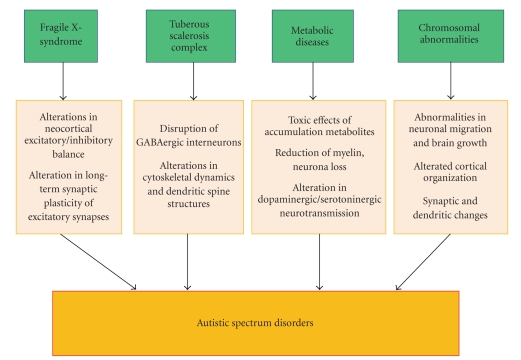
*Potential pathogenetic mechanisms of syndromic autism*. Several medical conditions associated with syndromic autism appear to influence and potentially disrupt neurodevelopmental processes, including brain growth, cortical connectivity, and neurotransmitters pathways. These neurobiological alterations likely affect the developmental trajectory of social behavior and communication during early stages of childhood and determine the different clinical phenotypes of ASD.

Several molecular pathways potentially involved in the disruption of neurodevelopmental trajectories during intrauterine or postnatal brain development may be associated with abnormal developmental processes, from neuronal migration and cortical organization to synaptic and dendritic conformation [69]. Furthermore, environmental factors, including maternal/intrauterine infections, exposure to toxins, and ossidative stress, may modify the underlying genetic substrate and leading to abnormalities in neuronal organization and cortical network development [70]. Figure 1 summarizes both genetic background and epigenetic factors involved in the pathogenesis of ASD, and explains how their multifactorial influence may be necessary for full expression of the autistic phenotype. Defined medical syndromes, chromosomal abnormalities and de novo copy number variations (CNVs) may account for 10% of ASD cases [71]. Figure 2 illustrates many different types of potential pathogenetic mechanisms responsible for the ASD phenotype in the most common medical conditions associated with autism. Widespread genetic testing would be expensive, time-consuming, and generally inappropriate due to the etiological complexity, while the appropriate use of genetic testing in subgroups of autistic patients showing particular clinical features is relevant to good clinical practice and may allow the identifications of new susceptibility variants. The advent of fluorescent in situ hybridization (FISH) techniques has expanded the list of chromosomal hot spots in autism. Individual FISH studies may be indicated in the confirmation of a clinically suspected condition [72], and in the evaluation of low functioning patients with an IQ <50 [73]. When dysmorphic features are present, it is reasonable to suspect chromosomal rearrangements even if the karyotype appears normal, and oligo-array-based CGH analysis is highly advisable in these cases [74]. Whole genome-scanning by array-based technology has detected copy-number variations (CNVs), which are copy-number changes involving a DNA fragment, and represent submicroscopic deletions or duplications that are undetectable at the routine cytogenetic analysis [75–78]. In conclusion, as etiologies of ASD are progressively discovered, the number of individuals with idiopathic autism will progressively shrink. The role of the neuropediatrician will be to understand the neurological basis of autism, and to identify more homogenous subgroups with specific biologic markers. Because autism represents an extremely heterogeneous group of disorders, a better understanding of underlying biological processes will lead to more targeted intervention approaches that can be designed for specific subtypes of autism.

## Figures and Tables

**Table 1:** Diagnosis and potential therapeutic approaches in some metabolic diseases associated with autism.

Metabolic diseases	Potential patogenetic mechanisms	Clinical features	Diagnosis	Therapetic options	Refs
Phenylketonuria	Low production of dopamine and serotonin. Toxic effects on the brain cells. Reduction of myelin.	Neonatal onset Autism, seizures, severe mental retardation, hyperactivity, EEG abnormalities and seizures, microcephaly, albinism (excessively fair hair and skin) or a tendency to hypopigmentation and eczema, “musty or mousy” odor of skin, hair, sweat, and urine.	Quantitative plasma amino acids analysis. Dosage of phenylpyruvic acid in urine.	Restricted diet + aminoacids administration.	[28, 29]
Adenylosuccinase deficit	Toxic effects of the accumulating succinyl purines on the brain.	Onset in the first year. Autistic phenotype, profound psychomotor retardation, epilepsy, hypotonia, peripheral hypertonia, failure to thrive. No dismorphic features.	Succinyl aminoimidazole, carboxamide riboside and succinyl adenosine in urine and cerebrospinal fluid.	Therapy with D-ribose.	[7, 30]
Smith-Lemli-Opitz syndrome	Neurosteroid deficiency. Alteration of neuroendocrine functions and disruption of the growth and development of many body systems.	Onset in infancy. Autism, mental retardation, sensory hyperreactivity, irritability, language impairment, sleep cycle disturbance, self-injurious behavior, microchepaly, hypotonia, syndactyly, hypogenitalism, malformations of the brain, lung, heart, and gastrointestinal tract.	Abnormal sterol pattern (low plasma and tissue cholesterol concentrations, and increased plasma and tissue 7-dehydrocholesterol reductase and its metabolite).	Cholesterol replacement therapy.	[31, 32]
Creatine deficiency syndromes	Neurotoxic effect of guanidinoacetate or other guanidine compounds.	Autistic phenotype, mental retardation, speech delay, epilepsy, extrapyramidal symptoms, progressive encephalopathy with muscular hypotonia, dyskinetic movements, developmental arrest/regression.	Blood and urinary concentration on creatine and guanidinoacetate, Brain magnetic resonance spectroscopy.	Oral creatine supplementation. Restriction of arginine and substitution of ornithine.	[33]

**Table 2:** Genetic syndromes associated with autism.

Syndrome	Gene(s) associated with the syndrome	Proportion of patients with an ASD that have the syndrome	Proportion of patients with the syndrome that have an ASD	Clinical signs	Refs
Fragile-X syndrome	FMR1	2–5%	20–40%	Mental retardation, long face with prominent ears, macroorchidism, social anxiety, sensory hypersensitivity, stereotypies, poor motor coordination, delayed speech development.	[56, 57]
Tuberous sclerosis	TSC1, TSC2	3–4%	43–86%	Epilepsy, mental retardation, specific learning disabilities, ADHD disorder, autistic spectrum disorders.	[58, 59]
15q duplication Angelman/Prader Will syndrome	UBE3A GABAr cluster	1–2%	>40%	Ataxia, language delay, epilepsy, mental retardation, repetitive movements, obsessive-compulsive symptoms.	[60]
16p11 deletion	PCKB1	1%	High	Developmental delay, distinct facial appearance, autism.	[46, 61]
22q deletion	SHANK3	1%	High	Speech and language disability, social impairment.	[62, 63]
2q37 deletion	KIF1A, GBX2	Unknown	50%	Developmental delay, mental retardation, hypotonia, hyperactivity, autistic traits, dysmorphic features (cleft palate, temporal bone abnormalities, hypoplastic lungs).	[64]
Joubert syndrome	*AHI1*	Unknown	40%	Partial/complete agenesis of the cerebellar vermis, ataxia, abnormalities of ocular movements, cognitive, and behavioral dysfunction.	[65]
Timothy syndrome	CACNA1C	Unknown	60–70%	Cardiac arrhythmia, long QT syndrome, mental retardation, and ASD.	[66]
Cortical dysplasia-focal epilepsy syndrome	CNTNAP2	Rare	70%	Seizures and language regression.	[67, 68]

**Table 3:** Candidate genes associated with autism.

Genes	Chromosomes	Proteins	Proteins' functions	Neurobiological abnormalities	Clinical phenotypes	Refs
NGL3 NGL4	Xq13.1 Xp22.3	Neuroligin 3/4.	Synaptic transmission, differentiation of synaptic contacts.	Synaptic or dendritic changes.	Autism with motor tics, Mild to severe autism, PDD-NOS, “regression” at disease onset, with a loss of initially-acquired social and verbal milestones, no dysmorphic features.	[79, 80]
SHANK3	22q13	Shank scaffolding proteins.	Master organizer of postsynaptic glutamatergic density.	Synaptic or dendritic changes.	Multiple developmental delays, dysmorphic features, autism with severe language/social deficits.	[81, 82]
MET/HGF	7q31	MET receptortyrosine kinase/hepatocyte growth factor.	Regulation of dendritic morphology and promoting neurite outgrowth.	Abnormalities in development of the cerebral cortex and cerebellum.	Autism, increased anxiety, seizures, immune, and gastrointestinal problems.	[83]
MECP2	Xq28	Methyl-CpG-binding protein 2.	Synapse maintenance and remodeling.	Synaptic or dendritic changes.	Rett syndrome with regression, mental retardation, microcephaly, stereotyped behaviors, epilepsy and breathing problems; verbal Rett variants.	[84]
HOXA1	7p15.3	Homeobox protein.	Regulation of brain growth.	Abnormalities of numbers of neurons or glia in the brain.	Mental retardation, autism and distinct clinical features (horizontal gaze abnormalities, focal weakness, hypoventilation, vascular malformations).	[85]
PTEN	10q23	phosphatase and tensin homologue.	Regulation of cells proliferation/differentiation.	Abnormalities in brain growth.	Macrocephaly, macrosomia, autism and developmental delay, increased risk of developing a variety of PTEN-related cancers in adulthood.	[86, 87]
